# Innovative Approach to Understanding Complex Neuroanatomy Through ‘Acting‐Out’, Immersive 3D Modelling

**DOI:** 10.1111/tct.70276

**Published:** 2025-12-08

**Authors:** Charlotte Kulow, Mara Sandrock

**Affiliations:** ^1^ Institut für Anatomie Medizinische Fakultät der Universität Leipzig Leipzig Germany

## Abstract

The challenge persists of engaging students in anatomy education, especially neuroanatomy. Conventional lectures often fail to accommodate the diverse learning preferences of students, leading to disinterest and stress. Innovative teaching methods, such as gamification and interactive learning, have shown promise. Recent advances, like 3D printing, card games and basic materials, have created more tactile models that enhance student engagement. Leipzig's anatomy department has developed a method called ‘Acting‐out’ to teach and understand the spinal tracts, autonomic nervous and limbic systems. The ‘Acting‐out’ method involves the collaborative creation of an enlarged neurological complex symbolising a particular aspect of structure, within which participants immerse themselves through role‐playing scenarios, embodying and personifying a specific part or nerve structure. This method employs immersive 3D models, enhancing spatial understanding, encouraging collaboration and critical thinking. Students physically embody anatomical structures. Our ‘Acting‐out’ method aligns with modern pedagogical principles. Physical activity enhances learning, while role play fosters deeper comprehension. Assigning roles and becoming structures provides unique perspectives, aiding memory retention. Peer teaching encourages reinforcement and cultivates a supportive environment. The ‘Acting‐out’ method's unconventional approach has succeeded in engaging students. By stepping outside of traditional bounds, educators can offer students enriching, transformative educational experiences that prepare them for the dynamic demands of their career.

## 1 What Is the Challenge?

One of the challenges facing anatomy educators today is to engage and motivate students to learn without pressure from grading and hierarchy. The teaching of neuroanatomy, in particular, can be a complex and challenging task for both educators and students. Whilst conventional didactic lectures remain prevalent [1, 2]. Research indicates that this approach may not consistently address the diverse needs of a high percentage of students [3]. Conventional methods fail to meet the individual needs of many students; this then promotes negative factors such as fear, concentration loss, lack of confidence, stress and ultimately the loss of interest in the course itself [4]. Unfortunately, many students and teachers view anatomy as a list of structures that needs to be learned by rote, devoid of intrinsic interest [5]. However, innovative teaching methods show that novel approaches to teaching anatomy or neuroanatomy, engage students enthusiastically. This often enhances learning [6, 7, 8]. The recent integration of gamification and interactive student‐led learning has emerged as a promising avenue for augmenting teaching efficacy and student engagement [9, 10]. Innovative smaller three‐dimensional models created with 3D printing [11] or basic materials like paper plates, pipe cleaners and plasticine clay aid in the teaching of spinal tracts [10, 12]. These models empower students to engage in tactile learning, facilitating hands‐on manipulation and self‐guided exploration. In this paper, we introduce a new interactive teaching method for mastering these complicated anatomical structures: the spinal tracts, autonomic nervous system and limbic systems. This method complements recently developed approaches described above [10, 12].

## 2 What Is the Proposed Solution?

Here in the Institute for Anatomy, Leipzig University, we often see first and second‐year medical students struggle to learn many new and challenging concepts. To address this, we created alternative teaching methods for learning anatomy and neuroanatomy electives. These electives accommodated up to 20 students per elective, with two electives offered per year. The primary goal of these classes is to offer medical students an alternative mode of learning. Within these electives, we created an ‘Acting‐out’ method. These ‘Acting‐out’ exercises were specifically designed for learning and understanding the spinal tracts, autonomic nervous system and limbic system. The ‘Acting‐out’ methodology constitutes a dynamic pedagogical strategy involving immersive 3D modelling. It enables students to visualise different structures, actively participate, understand spatial relationships, interact, collaborate, improve long‐term memory, work in a team and develop communication, examination, presentation and critical thinking skills [13]. Overall, the ‘Acting‐out’ method provides students with a range of benefits that can enhance their learning experiences. By using engaging methods like the ‘Acting‐out’ approach, educators can enhance students' learning experiences in neuroanatomy, making complex concepts more accessible and enjoyable [14].

Pedagogical strategy in involving immersive 3D modelling.

## 3 How Was the Solution Implemented?

First and second year medical students at the Institute for Anatomy, Leipzig University, participated in an elective on alternative teaching methods for anatomy and neuroanatomy. In total, 80 students over 2 years took part in these larger electives. Within each elective, 20 students were allotted 2.5 h for this specific ‘Acting out’ method. The resources used were either found at home/work or bought prior to the lectures. No additional support staff or tech team is necessary.

An elective on alternative teaching methods for anatomy and neuroanatomy.

### 3.1 Game 1: Parasympathetic and Sympathetic Nervous System (Figure 1)

**FIGURE 1:**
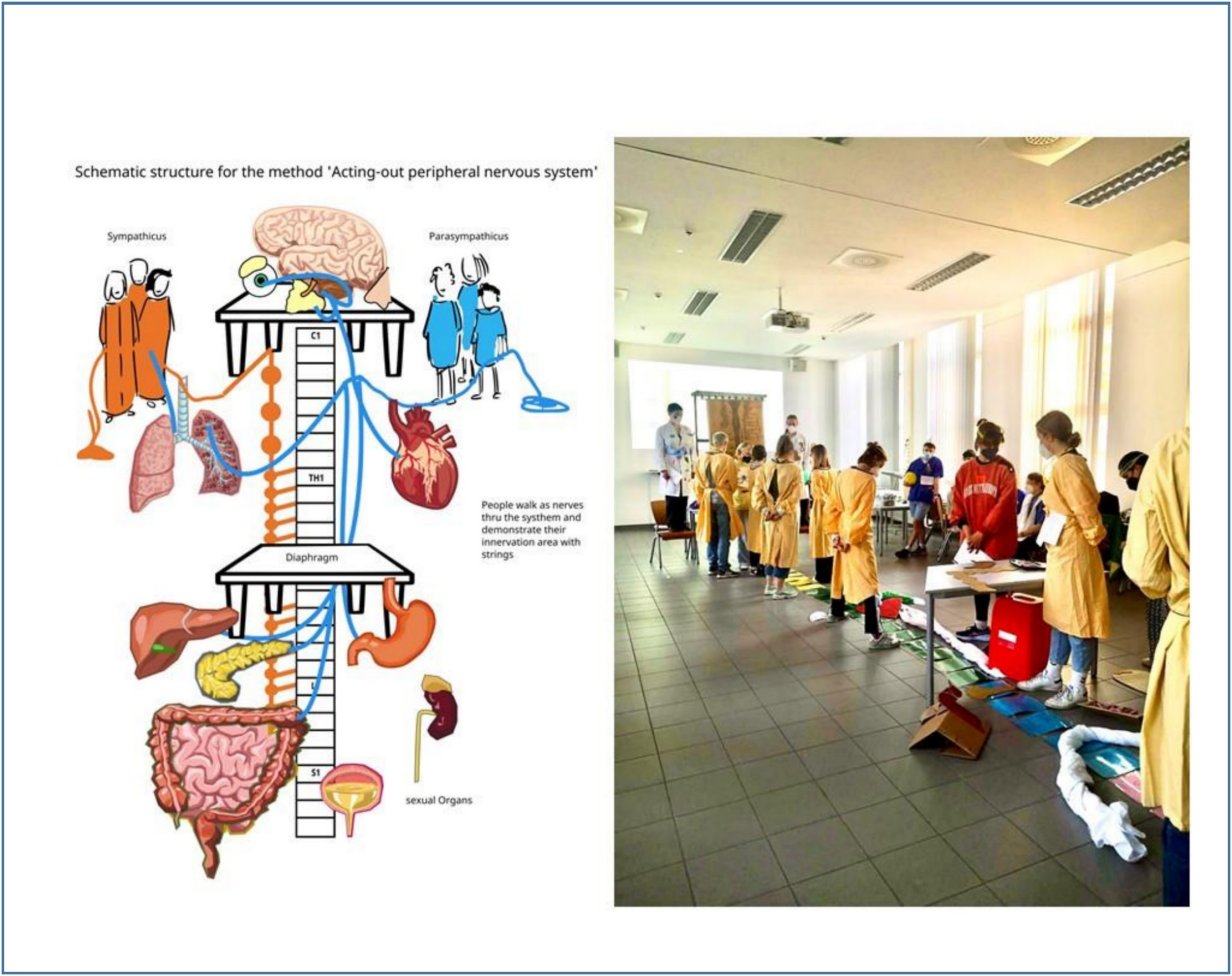
Setup of game 1.

#### 3.1.1 General Set‐Up

We used a large room equipped with chairs, tables and a blackboard. Before the class, we designed a spine layout using 26 coloured A4 pages to represent the five different sections of the spine (Figure 1). These pages were connected with string tags to form a long chain. A long cloth represents the aorta. Three red buckets symbolised the coeliac trunk, inferior mesenteric artery and superior mesenteric artery.

##### 3.1.1.1 The Students

To prepare for the session, each student was assigned a nerve or neural structure and asked to research key details to teach their peers. This can be done as a flipped classroom. During the session, the students had approximately 45–60 min to prepare, set up the room and create a costume for their assigned structure (Figure 1); this time can be reduced if organised prior to the lecture. The materials provided were adhesive paper, string, face paint, pipe cleaners in various colours, pens, paper, socks, tights and other crafting materials. Students who belonged to the parasympathetic system and sympathetic system wore contrasting coloured lab coats (e.g., blue for parasympathetic and yellow for sympathetic) to visually distinguish these systems.

**BOX 1 Materials needed.**

**Spinal Layout**: 26 coloured A4 pages connected with string tags

**Aorta Representation**: Long cloth

**Arterial Structures**: Three red buckets for the coeliac trunk, inferior mesenteric artery and superior mesenteric artery

**Organ Models**: Paper, cardboard and craft materials to construct organs such as eyes, ears, kidneys, bowels, liver, pancreas and sex organs, coloured accordingly and placed along the spinal pathway at correct anatomical heights.

**Nerve Depiction**: A large bag of coloured ropes (preferred over wool, as wool tends to be too thin and break easily).

**Autonomic systems:** contrasting lab coats or t‐shirts

##### 3.1.1.2 The Game

When everything was set up, the game began. The brains (left and right cortex) were the leaders and stood on a table for a greater overview. They were able to help position the overall scheme and movements. For example, the person playing cranial nerve III innervates the ciliary muscle and the sphincter pupillae. The student demonstrates the path of this nerve by walking with a string from the brainstem towards the eye, tying a knot for the ciliary ganglion and describing how it affects the eye (e.g., accommodation, pupillary constriction and dilation). Everyone is allowed to ask questions about pathways and functions, and the students involved are asked to teach and provide mnemonics or tell what they found interesting during their preparatory reading about the nerve. This method of play is used for all the nerves of the two systems, with each scenario including each pathway, target organ and function.

Students involved are asked to teach and provide mnemonics.

Different situations can be re‐enacted in the last part of the lesson, and special configurations can be clarified:

  Example 1 The sympathetic neck ganglia, which repeatedly causes confusion. The ascending branches leave the sympathetic chain within the thorax and pass through the cervical ganglia. A lesion can then be simulated at different heights. This is used as the explanation and visualisation of Horner's syndrome (miosis).
  Example 2 The course of the pelvic splanchnic nerves, which arise from the thorax. The student has to crawl through the diaphragm (under a table) to reach their target organs.
  Example 3 The shared innervation of excitation of the genitals. Using male anatomy as an example, it is easy to show that the parasympathetic nervous system is responsible for arousal and the sympathetic nervous system for ejaculation.

### 3.2 Game 2: Spinothalamic Tract and Dorsal Column–Central Nerve Pathways (Figure 2)

**FIGURE 2:**
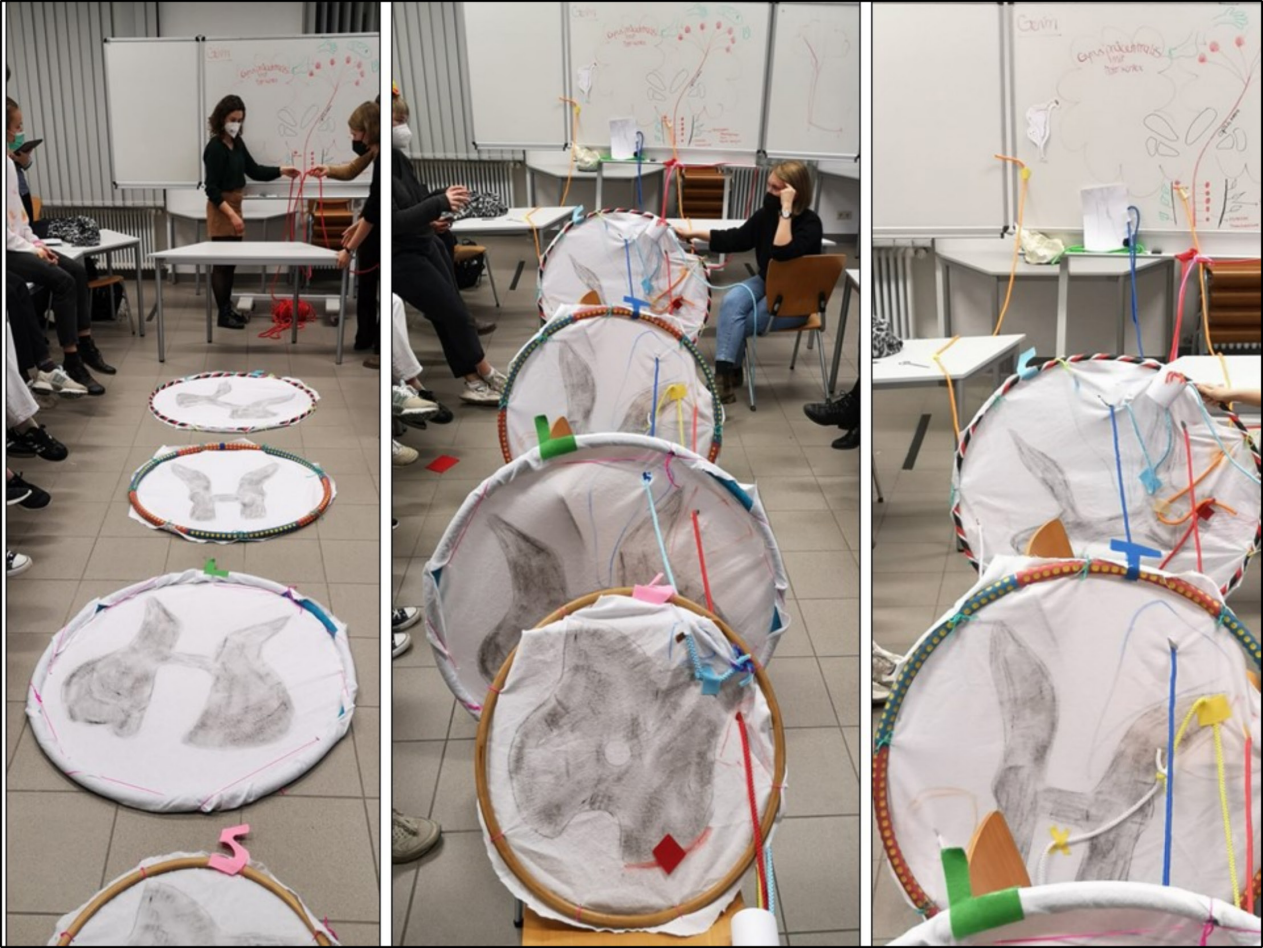
Setup of game 2.

#### 3.2.1 General Set‐Up

Using a large room, we provided four hula hoops of varying sizes, white cloth, coloured ropes, coloured sticky tape, crafting materials and a whiteboard. Students select the spinal cord at four different heights, paint and draw grey and white matter on different‐sized pieces of cloth, and attach them to the hula hoops. That takes a bit of ingenuity! The basic outline of the brain was drawn on a whiteboard, showing white and grey matter and as large as possible. Symbols representing the nerve nuclei were painted/marked on the relevant location. For example, beetroot (ruber in German) was used to depict the red nucleus, and was written in red and an associative image was drawn.

**BOX 2 Materials needed.**

**Spinal Layout**: Four hula hoops of varying sizes

**Spinal grey and white matter**: White cloth attached to hula hoops

**Nerve Depiction**: Coloured ropes

**Nerve nuclei**: Coloured sticky tape

**Brain outline**: Whiteboard

**Qualities of the structures:** extra craft materials

##### 3.2.1.1 The Students

The students were divided into four different groups. The first group represented the pyramidal tract, the second the extrapyramidal tract, the third the ascending pain and temperature and the fourth, fine touch sensibility.

The students should be given the opportunity to prepare for the content of their track within their small groups before coming together as a larger group. To achieve this, they initially constructed a normal‐sized 3D model solely representing their assigned pathway, with the use of paper plates, paper cut outs and pipe cleaners. The goal was to gain a comprehensive understanding of the route, intersections and functions and to represent these aspects visually using symbols and colour coding.

##### 3.2.1.2 The Game

The students dress up using hats or badges to enhance memory recall of who they represent. They then use different coloured ropes to follow the pathways, walking, talking and acting their way through the pathways (Figure 2). Associative symbols are used for qualities of the structures, for example, pyramidal path: pyramid‐shaped headgear/hats of the actors; pain and temperature sensitivity: pain associated with a painted black eye of the actor. In the shared setting, the task then consisted of following the nerve track with the help of coloured ropes and breaking through the corresponding points in the drawn spinal cord tissue attached to the hula hoops. Crossings should be indicated, and structures passed through should be named concisely. Symbols, memory aids, mnemonics and conspicuous objects can be added as needed and made available to the other students.

The students dress up using hats or badges to enhance memory recall

Following these four presentations, clinical cases could then be applied to what had been learned, and symptoms, models and explanations could be played through together, for example, paraplegia or Brown‐Sequard syndrome.

### 3.3 Game 3: Papez‐Neuron Circuit (“I pack my bag, and in it, I put …”) (Figure 3)

**FIGURE 3:**
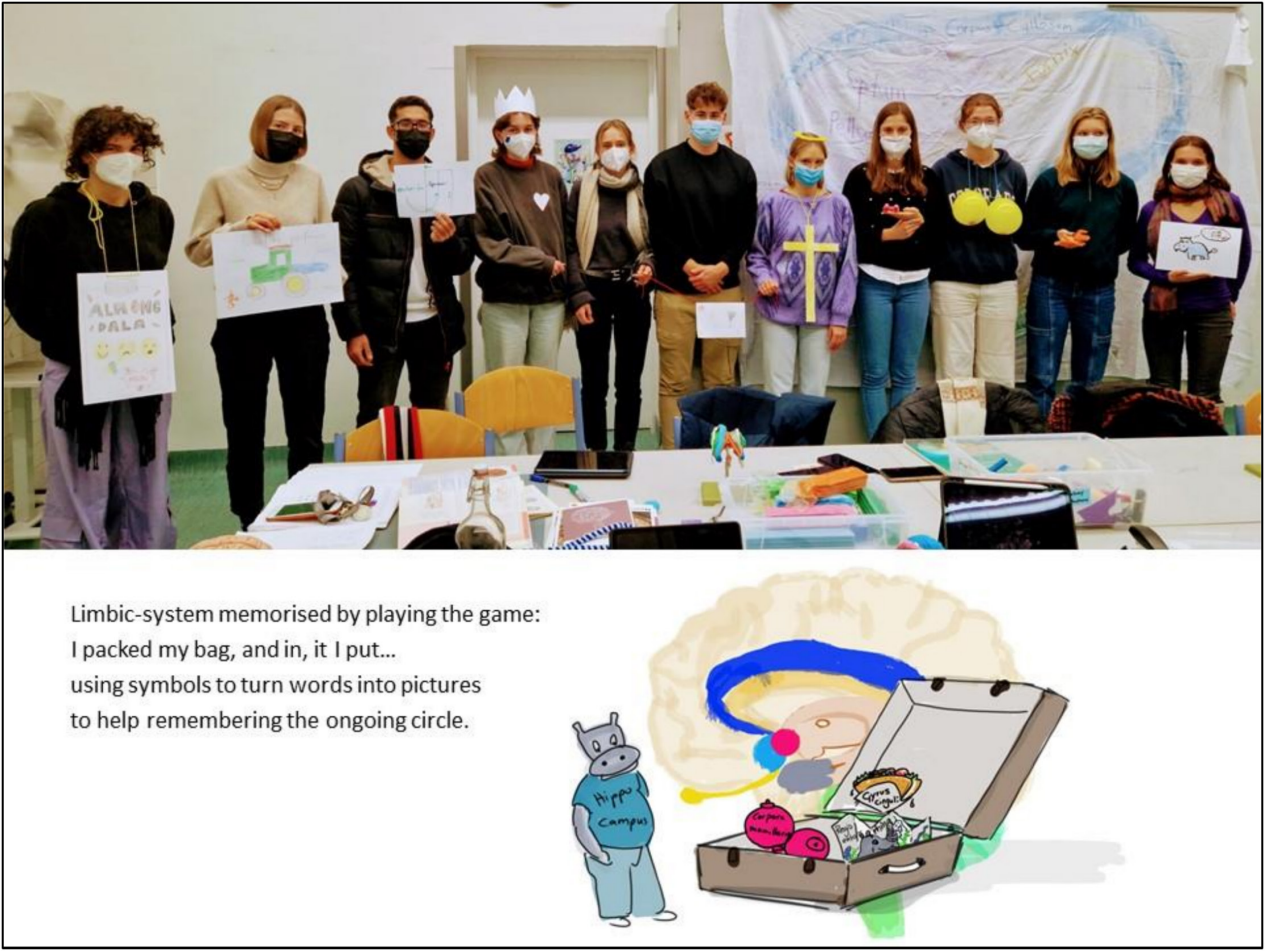
Setup of game 3.

This is an adaptation of a children's memory game. The first player begins by saying, ‘I pack my bag, and in it, I put …’. The next player then recalls the first player's item and adds their own. Players continue taking turns, adding objects to the suitcase, until someone forgets what has been packed. There are various variations of this game, but it is essentially a memory game (Figure 3).

#### 3.3.1 The students

The Papez‐neuron circuit, as a part of the limbic system, is a circle with different stations. So we thought the game ‘I pack my bag, and in it, I put …’ could be applied to it because it goes round and round and needs to be remembered in a specific sequence.

**BOX 3 Materials needed.**

**The brain:** large white piece of fabric and coloured pens or chalk

**Individual anatomical parts:** crafting materials

Every participant represented one station or pathway of the circle. The students had 15–25 min to prepare their role, using online or textbook‐based research. They should find out the part's influence on the system, its function and how it can be implemented as an image. They were encouraged to either dress up, draw a picture or create an analogy or word association that would make their structure more memorable. For example, a student responsible for the fornix used ‘phoenix’ as the visualisation of the word and explained that the phoenix was flying over to the mammillary body, describing the path as a bow over the diencephalon.

When everyone was prepared, we stood in a circle. The amygdala started and said: ‘I am the amygdala, Amy, with an Almond (almond‐shaped nucleus); my function is …’. Then the next person took over and said: ‘You are the amygdala, Amy with an Almond …. And I am the hippocampus, the hippo, my function is …’. The round proceeded further, with each student always repeating what was said before, starting with the first person (Amy) (Figure 3).

When the round is finished, it can be repeated as often as necessary.

For better knowledge retention, it is important to have the visuals as noticeable as possible to increase the fun elements and conspicuousness of structures. For example, the mammillary body could be represented by balloons with a drawn nipple.

## 4 What Lessons Were Learned That Are Relevant to the Wider Global Audience

The vast amount of material needing to be understood in the area of neuroanatomy, in particular, can be overwhelming, leading students to be fearful of the subject [15]. Traditional pedagogical approaches to teaching neuroanatomy have been didactic in nature and often difficult to comprehend. Additionally, the constrained time frame of medical school's curricula adds another layer of complexity to the equation. Therefore, finding ways of teaching a vast number of students with diverse learning preferences is a challenge. In response to these challenges there is a growing recognition amongst educators to support the use of multimodal teaching methods over unimodal approaches. Acknowledging the uniqueness of individuals, educators are actively embracing more diverse learning opportunities. Notably, the emergence of game‐based methods as demonstrated by Chytas et al. [9] holds the promise of not only enhancing comprehension but also rekindling students' enthusiasm. Neurobiological and educational research supports the idea that gamification enhances memory retention through dopaminergic reward pathways (Box 4) [16]. Multisensory methods strengthen recall via cross‐modal neural integration, aligning with the idea that our blended pedagogical and andragogical approach fosters intrinsic learning and self‐directed application (Box 4) [17].

In this paper, we have described our own multimodal approach to teaching a small but relevant portion of the neurological system. We have expanded on 3D modelling to make it immersive, an enhancement of existing 3D neuroanatomy modelling for the spinal tracts [11]. Research by Estevez et al. [12] underlines the heightened efficacy of 3D modelling compared with 2D brain slice illustrations, amplifying the educational impact. The ‘Acting‐out’ method also combines various teaching techniques, effectively engaging all students (Box 4). Through collective participation in the system, students become immersed in the learning experience.

Through collective participation in the system, students become immersed in the learning experience.

Box 4 **Teaching techniques used.**

**Table:** 

	Benefits and research
Physical engagement	Facilitating neurogenesis in the hippocampus, a neural structure strongly relating to memory [16, 18]
Embodied cognition—learn by doing	Our physical bodies are actively linked to our interactions. Senses and movements can influence how we think and thus learn or retain knowledge—sensory and motor [17].
Role play	A deeper cognitive link to the material being learned when students engage in role play [19]. Personalise a structure to make it more memorable through active learning, movement, emotions and symbolism [20].
Memory hooks—Loci method	Memory aids or memory hooks are an invaluable way to learn and retain knowledge. Visual mnemonics, such as the loci method, have been shown to be an effective tool in learning, retaining and recalling complex medical concepts [21]
Peer to peer teaching	Peer teaching itself serves as an effective tool for fostering learning and knowledge retention. When students prepare to teach, they are prompted to construct their own learning program, enabling them to effectively explain concepts to their fellow learners [22, 23].
Presentation skills	Promotes a whole group dynamic where the room functions as a stage, ensuring that no individual feels exposed in front of the class.

From personal observation, the alternative methods offered for learning anatomy and neuroanatomy electives seem to attract students who are more inclined towards alternative learning approaches, resulting in a self‐selection process. However, even students who initially join out of necessity have reported positive experiences and personal growth. Our cohort ranged in age from 18 to 30, which indicates this method has potential use for all ages. This method is scalable and adaptable to other anatomical systems, locations and environments as the resources can be minimal.

As lecturers, we also encountered challenges with varying student moods and scepticism, but overall the feedback was positive. This response is not uncommon for new teaching methods; however, it must be considered during implementation [24]. Certainly, this extroverted method may also not align with the comfort zone of all educators, necessitating confidence and a willingness to experiment.

Incorporating this method into an already demanding curriculum can also be a challenge, particularly given the constraints of time, but by incorporating a flipped classroom this time can be reduced [25] Nevertheless, we would advocate that this approach holds immense value to those students struggling to grasp the complexities of these concepts. By incorporating alternative and innovative methods in teaching, we show that educators can provide students with diverse learning experiences that may initially appear unconventional [26]. Additionally, exposing students to these different approaches broadens their perspective and encourages critical thinking [16], positively impacting their overall educational journey and ultimately enhancing their skills as future clinicians.

Positively impacting their overall educational journey and ultimately enhancing their skills as future clinicians.

## Author Contributions

**Charlotte Kulow:** conceptualization, methodology, investigation, visualization, writing – review and editing, writing – original draft. **Mara Sandrock:** conceptualization, methodology, investigation, writing – original draft, visualization, writing – review and editing.

## Funding

The authors received no specific funding for this work.

## Conflicts of Interest

The authors declare no conflicts of interest.

## Data Availability

Data sharing is not applicable to this article as no datasets were generated or analysed during the current study.
